# Crystal structure of 1-ethyl-3-(2-oxo-1,3-di­thiol-4-yl)quinoxalin-2(1*H*)-one

**DOI:** 10.1107/S2056989018007892

**Published:** 2018-06-08

**Authors:** Nicolas Chrysochos, Carola Schulzke

**Affiliations:** a Institut für Biochemie, Ernst-Moritz-Arndt-Universität Greifswald, 4 Felix-Hausdorff-Strasse, 17487 Greifswald, Germany

**Keywords:** crystal structure, quinoxalin, ene-di­thio­carbonate, carbonodi­thio­ate, hydrogen bonding inter­action, molybdopterin

## Abstract

The title compound **I**, C_13_H_10_N_2_O_2_S_2_, crystallizes in the monoclinic space group *C*2/*c* with eight mol­ecules in the unit cell. Excluding for the ethyl substituent, the mol­ecule of **I** adopts a nearly coplanar conformation (r.m.s. deviations is 0.058 Å), which is supported by the intra­molecular C—H⋯O hydrogen-bonding inter­action between the two ring systems [C⋯O = 2.859 (3) Å]. In the crystal, the mol­ecules form dimeric associates *via* two bifurcated C—H⋯O hydrogen-bonding inter­actions between an ene hydrogen atom and a carbonyl functional group of an adjacent mol­ecule [C⋯O = 3.133 (3) Å] and *vice versa*. The crystal structure is further stabilized by a three-dimensional network of weak hydrogen bonds between one mol­ecule and six adjacent mol­ecules as well as offset π–π stacking. The combination of the quinoxaline 2(1*H*)-one moiety with the di­thio­carbonate moiety extends the aromaticity of the quinoxaline scaffold towards the substituent as well as influencing the π-system of the quinoxaline. The title compound is the direct precursor for a di­thiol­ene ligand mimicking the natural cofactor ligand molybdopterin.

## Abstract 2

A new quinoxalin-derived ene-di­thio­carbonate was synthesized and structurally analysed. The synthetic procedure and the compound’s spectroscopic and analytical characterization are reported.

## Chemical context   

Non-innocent di­thiol­enes and their role as inter­esting ligand systems were discovered in the early 1960s. As a result of their unusual redox and structural characteristics and those of their metal complexes, they immediately attracted considerable scientific inter­est (Schrauzer & Mayweg, 1962 ▸). Initially, di­thiol­ene systems were studied predominantly in the context of electronic and photonic conductors (Wudl *et al.*, 1972 ▸; Ferraris *et al.*, 1973 ▸). Later, metal di­thiol­ene complexes found application in the purification and separation of olefins (Wang & Stiefel, 2001 ▸). In the early 1980s Rajagopalan and co-workers discovered and characterized the natural molybdopterin ligand (mpt) in the active sites of enzymes. Mpt is present in nearly all molybdenum enzymes and all tungsten enzymes and binds the respective central metal by a di­thiol­ene moiety. As these enzymes are ubiquitous to all kingdoms of life, this brought di­thiol­ene chemistry again to the focus of scientific attention (Johnson *et al.*, 1980 ▸; Johnson & Rajagopalan, 1982 ▸; Kramer *et al.*, 1987 ▸). Quinoxaline constitutes a widely exploited platform in the development of pharmaceuticals (Shi *et al.*, 2018 ▸). The title compound is a di­thiol­ene ligand precursor, which can be used for the synthesis of molybdo­pterin cofactor model complexes bearing quinoxaline substit­uents. The target di­thiol­ene ligand replicates the pyrazine moiety of mpt in its half-reduced form and in addition contains an oxofunction in the position of the pyran ring of the natural product. By itself it is an inter­esting example of an extended π-system involving (by resonance) three different heteroatoms (N, O and S).
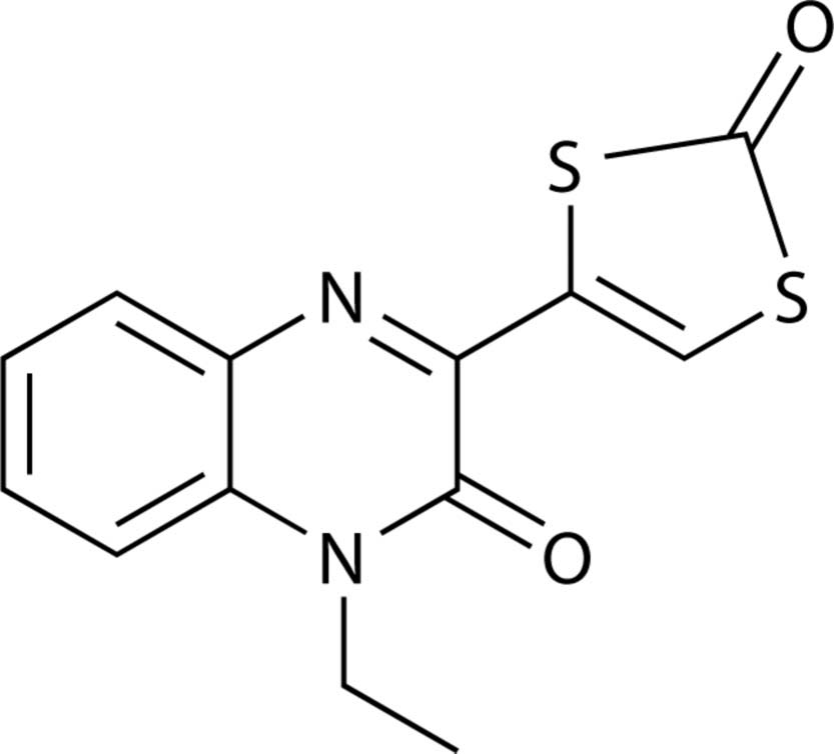


## Structural commentary   

**Figure 1:**
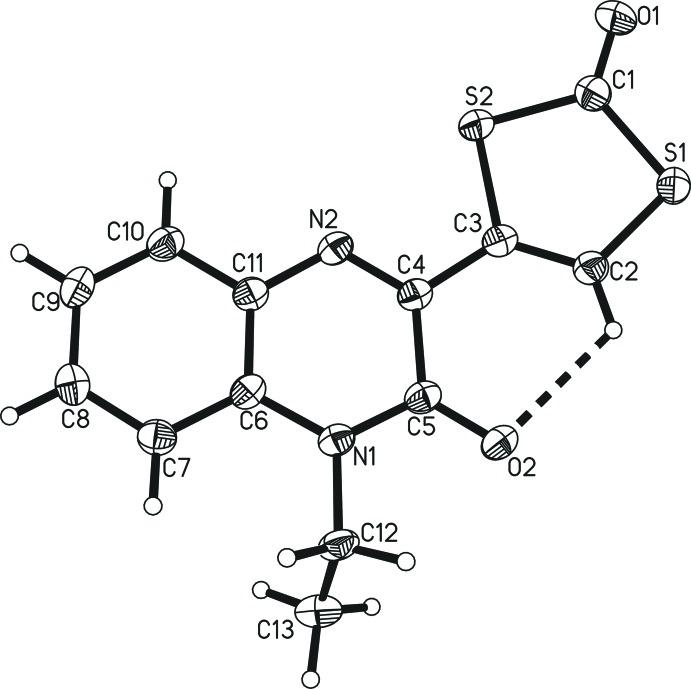
The mol­ecular structure of 1-ethyl-3-(2-oxo-1,3-di­thiol-4yl-)quinoxalin-2(1*H*)-one showing the atom labelling, 50% probability displacement ellipsoids and the intra­molecular non-classical hydrogen bond (dashed line).

The title mol­ecule **I** crystallizes in the monoclinic space group *C*2/*c* with *Z* = 8. The quinoxaline ring system [C4–C11, N1, N2; largest deviation from plane = 0.041 (2) Å for C5] and the di­thiol­ene ring [C1–C3, S1, S2; largest deviation from plane = 0.012 (1) Å for C3)], which are connected by the C3—C4 bond [length = 1.465 (3) Å], are essentially coplanar, with an angle of only 4.89 (12)° between the two planes (Fig. 1 ▸). This planitarity is supported by intra­molecular hydrogen bonding between the di­thiol­ene hydrogen atom and the quinoxaline carbonyl oxygen atom [C2—H2⋯O2 with *D*⋯*A* = 2.859 (3) Å; Table 1 ▸]. Only the alkyl substituent C12—C13 subtends out of the planar geometry with an N1—C12—C13 torsion angle of 112.78 (18)°. While the N1—C12 bond [amine nitro­gen and ethyl substituent; 1.475 (3) Å] is of explicit single-bond character, all other N—C distances are decidedly shorter, ranging from 1.296 (3) Å for the, according to the chemical structure, double bond of imine nitro­gen (N2=C4) to 1.392 (3) Å for the amine nitro­gen-to-benzene ring formal single bond (N1—C6). The longest C—C bond of the benzene ring is the one that is shared with the N-heterocycle [C6—C11, 1.411 (3) Å]. This, together with the adjacent N—C bonds [N1—C6, 1.392 (3) Å and C11—N2, 1.374 (3) Å] being significantly shorter than single bonds indicates resonance throughout the entire quinoxaline substituent. The C=O bond [1.212 (3) Å] of the carbonodi­thio­ate moiety is slightly shorter than the carbonyl C=O bond [1.232 (3) Å] of the quinoxaline substituent, suggesting that the latter might be involved to a small extent in resonance effects of the π-system, whereas the former is not. The C2=C3 double bond of the ene-di­thio­carbonate moiety is at 1.341 (3) Å slightly longer than the average value for C=C double bonds of 1.331 (9) Å (Allen *et al.*, 1995 ▸), which again may be due to participation in resonance effects throughout the entire mol­ecule. The deviation from the average value of 1.751 (17) Å for S—Csp^2^ bonds (Allen *et al.*, 1995 ▸) of the S1—C2 bond [1.724 (2) Å] is substantial enough to suggest that the resonance effects extend up to this bond to which partial double-bond character can be assigned. All other C—S bonds concur with typical single bonds.

**Figure 2:**
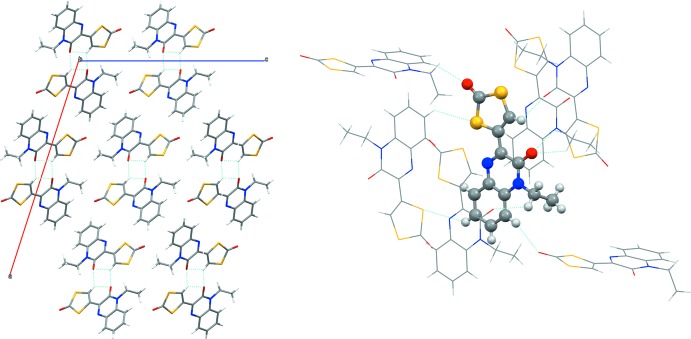
Crystal packing and intra- and inter­molecular hydrogen-bonding inter­actions yielding the dimeric associates viewed along *b* (left) and hydrogen bonding contacts of an individual mol­ecule to six adjacent mol­ecules (right) (*Mercury*; Macrae *et al.*, 2006 ▸).

By bidirectional inter­molecular hydrogen bonding, the title compound crystallizes as dimeric associate with the same donor and acceptor roles for both monomers [C2—H2⋯O2(−*x*, −*y* + 2, −*z* + 1) and O2⋯H2—C2(−*x*, −*y* + 2, −*z* + 1); *D*⋯*A* = 3.133 (3) Å]. Here, the exact same atoms are involved as in the intra­molecular hydrogen bond mentioned above. The respective hydrogen atom H2 is therefore bound to the ene carbon atom C2 and hydrogen bonded to the carbonyl oxygen (O2) of the quinoxaline moiety of the same mol­ecule as well as that of the adjacent mol­ecule (Fig. 2 ▸, left). There are only two crystal sructures of very closely related systems reported in the literature. In one (*A*), the quinoxalin substituent of the present mol­ecule is replaced by a coumarine (Ghosh *et al.*, 2016 ▸). In the other (*B*), the ene-di­thio­carbonate is replaced by an amino­thia­zole (Mamedov *et al.*, 2005 ▸). The metrical parameters in both structures are, as far as comparable, very similar to the ones observed here. Notable differences comprise (i) a slightly stronger resonance involvement of the thia­zole in *B* compared to the ene-di­thio­carbonate while the quinoxalin carbonyl and amine functions are embraced to a lesser extent and (ii) an overall weaker resonance in *A*, in which the benzene ring C—C distances are all very similar (*i.e*. strongly resonant) whereas all other distances are of more pronounced single- and double-bond character and of less aromatic character.

## Supra­molecular features   

In the crystal, the associated dimers are linked by (partly rather weak) C—H⋯O and C—H⋯S hydrogen-bonding inter­actions, forming a three-dimensional network (Fig. 2 ▸, Table 1 ▸). In the three-dimensional network, each mol­ecule forms hydrogen-bonding inter­actions to six surrounding mol­ecules. These are donor inter­actions involving C2 [C2—H2⋯O2(−*x*, −*y* + 2, −*z* + 1); *D*⋯*A* = 3.133 (3) Å], C7 [C7—H7⋯O1(*x*, −*y* + 1, *z* + 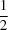); *D*⋯*A* = 3.272 (3) Å], C9 [C9—H9⋯S2(−*x* + 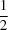, −*y* + 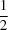, −*z* + 1); *D*⋯*A* = 3.652 (3) Å], C12 [C12—H12*A*⋯O2(*x*, *y* − 1, *z*); *D*⋯*A* = 3.367 (3) Å] and acceptor inter­actions involving S2 [S2⋯H9—C9(−*x* + 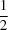, −*y* + 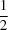, −*z* + 1)], O2 [O2⋯H2—C2(−*x*, −*y* + 2, −*z* + 1), O2⋯H12*A*—C12(*x*, *y*–1, *z)*] and O1 [O1⋯H7—C7(*x*, −*y* + 1, *z* − 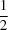)]. Even though there are coplanar alignments of layers, only offset π–π stacking was observed with centroid–centroid distances of 3.587 (3) Å between the benzene ring of one mol­ecule and the pyrazine ring of a mol­ecule in the layer above or below.

## Synthesis and crystallization   

The title compound, 1-ethyl-3-(2-oxo-1,3-di­thiol-4yl-)quinoxalin-2(1*H*)-one was synthesized based on a reported literature procedure (Mamedov *et al.*, 2005 ▸). The compound was synthesized in five steps starting from *o*-phenyl­enedi­amine. The last step in the synthetical pathway was carried out *via* an acid-catalysed Tchugaeff ring closure reaction, which led to the formation of the di­thiol­ene ring.

**Synthesis of 1-ethyl-3-(2-oxo-1,3-di­thiol-4yl-)quinoxalin-2(1** ***H*** **)-one:** To a solution of *S*-2-(4-ethyl-3-oxo-4-di­hydro­quinoxalin-2-yl)-2-oxo-ethyl *o*-isopropyl carbonodi­thio­ate (11.180 g, 31.9 mmol) in 250 ml DCM/Et_2_O 1:1 at ambient temperature, H_2_SO_4_ (25.50 ml) was added. The reaction mixture was stirred at room temperature for 2h. After that, the reaction was quenched by addition of 250 ml of ice and the mixture was stirred for 30 min. The organic phase was washed with brine and water 3 × 250 ml. The solvent was reduced to 10 ml *in vacuo* and the greenish precipitate was filtered off and washed on the filter with cold acetone 3 × 50 ml. The title compound was obtained as a greenish-white powder. Single crystals suitable for X-ray analysis were obtained by slow diffusion of solvents with chloro­form and Et_2_O Yield: 1.85g (20%).

^1^H NMR (300MHz, CD_3_Cl) δ 8.79 ppm (*s*, 1H), 7.87 ppm (*m*, 1H), 7.61 ppm (*m*, 1H), 7.4 ppm (*m*, 1H), 4.39 ppm (*q*, *J* = 7.2Hz, 2H), 1.42 ppm (*t*, *J* = 7.3Hz, 3H). ^13^C NMR (300MHz, CD_3_Cl) δ 152.54 ppm, 144.75 ppm, 133.25 ppm, 132.58 ppm, 131.14 ppm, 130.32 ppm, 126.98 ppm, 124.00 ppm, 113.47 ppm, 37.47 ppm, 12.20 ppm. IR (KBr pellet): (ν cm^−1^) = 3495 (*br*), 1734 (*w*), 1646 (*sst*), 1601 (*st*), 1579 (*st*), 1535 (*st*), 1463 (*st*), 1383 (*w*), 1280 (*st*), 1248 (*w*), 1216 (*w*), 1173 (*st*), 1128 (*w*), 1087 (*w*), 1045 (*w*), 950 (*w*), 892 (*st*), 868 (*w*), 825 (*st*), 785 (*w*), 758 (*st*), 631 (*w*), 554 (*w*), 529 (*w*), 467 (*w*), 432 (*w*). APCI–MS (*m*/*s*) = 291 (*M* ^+^ + H^+^). Analysis calculated for C_13_H_10_N_2_O_2_S_2_: C, 53.78; H 3.47; N 9.65; S 22.09. Found: C, 53.41; H 3.25; N 9.86; S 22.32.

## Refinement   

Crystal data, data collection and structure refinement details are summarized in Table 2 ▸. The hydrogen atom of the di­thiol­ene unit (H2) was refined freely without any constraints or restraints. All other C-bound hydrogen atoms were attached in calculated positions and treated as riding: C—H = 0.98 Å with *U*_iso_(H) = 1.5*U*_eq_(C) for the methyl group, C—H = 0.99 Å with *U*_iso_(H) = 1.2*U*_eq_(C) for the methyl­ene group and C—H = 0.95 Å with *U*_iso_(H) = 1.2*U*_eq_(C) for the aromatic atoms.

## Supplementary Material

Crystal structure: contains datablock(s) I. DOI: 10.1107/S2056989018007892/kq2022sup1.cif

Structure factors: contains datablock(s) I. DOI: 10.1107/S2056989018007892/kq2022Isup2.hkl

Supporting information file. DOI: 10.1107/S2056989018007892/kq2022Isup3.cml

CCDC reference: 1845671

Additional supporting information:  crystallographic information; 3D view; checkCIF report

## Figures and Tables

**Table 1:** Hydrogen-bond geometry (Å, °)

*D*—H⋯*A*	*D*—H	H⋯*A*	*D*⋯*A*	*D*—H⋯*A*
C2—H2⋯O2	0.95 (2)	2.30 (3)	2.859 (3)	117.2 (19)
C2—H2⋯O2^i^	0.95 (2)	2.44 (2)	3.133 (3)	129 (2)
C7—H7⋯O1^ii^	0.95	2.51	3.272 (3)	138
C9—H9⋯S2^iii^	0.95	2.99	3.652 (3)	128
C12—H12*A*⋯O2^iv^	0.99	2.69	3.367 (3)	126

Symmetry codes: (i) 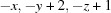; (ii) 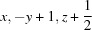; (iii) 
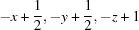
; (iv) 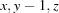.

**Table 2:** Experimental details

Crystal data	
Chemical formula	C_13_H_10_N_2_O_2_S_2_
*M*_r_	290.35
Crystal system, space group	Monoclinic, *C*2/*c*
Temperature (K)	170
*a*, *b*, *c* (Å)	25.531 (6), 4.8522 (10), 20.861 (4)
β (°)	107.81 (3)
*V* (Å^3^)	2460.4 (10)
*Z*	8
Radiation type	Mo *K*α
μ (mm^−1^)	0.43
Crystal size (mm)	0.48 × 0.46 × 0.04

Data collection	
Diffractometer	Stoe IPDS 2T
Absorption correction	Numerical (*X-RED32* and *X-SHAPE*; Stoe & Cie, 2010 ▸)
*T*_min_, *T*_max_	0.680, 0.887
No. of measured, independent and observed [*I* > 2σ(*I*)] reflections	10078, 2599, 2052
*R*_int_	0.107
(sin θ/λ)_max_ (Å^−1^)	0.633

Refinement	
*R*[*F* ^2^ > 2σ(*F* ^2^)], *wR*(*F* ^2^), *S*	0.043, 0.113, 1.03
No. of reflections	2599
No. of parameters	177
H-atom treatment	H atoms treated by a mixture of independent and constrained refinement
Δρ_max_, Δρ_min_ (e Å^−3^)	0.39, −0.44

Computer programs: *X-AREA* (Stoe & Cie, 2010 ▸), *SIR92* (Altomare *et al.*, 1994 ▸), *SHELXL2016/6* (Sheldrick, 2015 ▸), *XP* in *SHELXTL* (Sheldrick, 2008 ▸), *Mercury* (Macrae *et al.*, 2006 ▸) and *CIFTAB* (Sheldrick, 2013 ▸).

## supplementary crystallographic information

### Crystal data

**Table:**

C_13_H_10_N_2_O_2_S_2_	*F*(000) = 1200
*M**_r_* = 290.35	*D*_x_ = 1.568 Mg m^−^^3^
Monoclinic, *C*2/*c*	Mo *K*α radiation, λ = 0.71073 Å
*a* = 25.531 (6) Å	Cell parameters from 10798 reflections
*b* = 4.8522 (10) Å	θ = 3.4–53.5°
*c* = 20.861 (4) Å	µ = 0.43 mm^−^^1^
β = 107.81 (3)°	*T* = 170 K
*V* = 2460.4 (10) Å^3^	Platelet, green
*Z* = 8	0.48 × 0.46 × 0.04 mm

### Data collection

**Table:**

Stoe IPDS2T diffractometer	2599 independent reflections
Radiation source: fine-focus sealed tube	2052 reflections with *I* > 2σ(*I*)
Detector resolution: 6.67 pixels mm^-1^	*R*_int_ = 0.107
ω scans	θ_max_ = 26.7°, θ_min_ = 1.7°
Absorption correction: numerical (X-Red32 and X-Shape; Stoe & Cie, 2010)	*h* = −32→32
*T*_min_ = 0.680, *T*_max_ = 0.887	*k* = −6→6
10078 measured reflections	*l* = −26→23

### Refinement

**Table:**

Refinement on *F*^2^	0 restraints
Least-squares matrix: full	Hydrogen site location: mixed
*R*[*F*^2^ > 2σ(*F*^2^)] = 0.043	H atoms treated by a mixture of independent and constrained refinement
*wR*(*F*^2^) = 0.113	*w* = 1/[σ^2^(*F*_o_^2^) + (0.0557*P*)^2^ + 1.3022*P*] where *P* = (*F*_o_^2^ + 2*F*_c_^2^)/3
*S* = 1.03	(Δ/σ)_max_ < 0.001
2599 reflections	Δρ_max_ = 0.39 e Å^−^^3^
177 parameters	Δρ_min_ = −0.44 e Å^−^^3^

### Special details

**Table:**

Geometry. All esds (except the esd in the dihedral angle between two l.s. planes) are estimated using the full covariance matrix. The cell esds are taken into account individually in the estimation of esds in distances, angles and torsion angles; correlations between esds in cell parameters are only used when they are defined by crystal symmetry. An approximate (isotropic) treatment of cell esds is used for estimating esds involving l.s. planes.

### Fractional atomic coordinates and isotropic or equivalent isotropic displacement parameters (Å^2^)

**Table:**

	*x*	*y*	*z*	*U*_iso_*/*U*_eq_	
S1	0.06586 (2)	1.17332 (13)	0.38431 (3)	0.02924 (17)	
S2	0.16189 (2)	0.80790 (12)	0.43550 (3)	0.02882 (17)	
O1	0.14629 (7)	1.1274 (4)	0.32955 (8)	0.0379 (4)	
O2	0.04198 (6)	0.7134 (3)	0.56260 (8)	0.0296 (4)	
N1	0.09576 (7)	0.3591 (4)	0.61708 (9)	0.0233 (4)	
N2	0.16734 (7)	0.4593 (4)	0.54239 (9)	0.0242 (4)	
C1	0.12850 (9)	1.0512 (5)	0.37418 (11)	0.0290 (5)	
C2	0.06873 (9)	0.9742 (5)	0.45374 (11)	0.0258 (5)	
C3	0.11163 (9)	0.8032 (5)	0.47709 (11)	0.0237 (4)	
C4	0.12261 (8)	0.6022 (5)	0.53199 (10)	0.0229 (4)	
C5	0.08324 (8)	0.5692 (5)	0.57104 (10)	0.0236 (4)	
C6	0.14444 (8)	0.2091 (5)	0.63101 (11)	0.0231 (4)	
C7	0.15922 (9)	0.0081 (5)	0.68143 (11)	0.0263 (5)	
H7	0.135972	−0.028724	0.708326	0.032*	
C8	0.20757 (9)	−0.1364 (5)	0.69197 (11)	0.0278 (5)	
H8	0.217320	−0.273223	0.726147	0.033*	
C9	0.24248 (9)	−0.0852 (5)	0.65328 (12)	0.0296 (5)	
H9	0.275622	−0.187017	0.661099	0.036*	
C10	0.22885 (9)	0.1124 (5)	0.60399 (11)	0.0273 (5)	
H10	0.252595	0.147249	0.577626	0.033*	
C11	0.17997 (9)	0.2633 (5)	0.59235 (11)	0.0237 (4)	
C12	0.05590 (9)	0.2972 (5)	0.65349 (12)	0.0269 (5)	
H12A	0.054369	0.095209	0.659446	0.032*	
H12B	0.018905	0.359079	0.625995	0.032*	
C13	0.07039 (10)	0.4352 (6)	0.72202 (12)	0.0353 (6)	
H13A	0.108738	0.394349	0.747326	0.053*	
H13B	0.046176	0.365385	0.746832	0.053*	
H13C	0.065600	0.634974	0.716145	0.053*	
H2	0.0387 (10)	0.987 (5)	0.4716 (12)	0.028 (6)*	

### Atomic displacement parameters (Å^2^)

**Table:**

	*U*^11^	*U*^22^	*U*^33^	*U*^12^	*U*^13^	*U*^23^
S1	0.0263 (3)	0.0320 (3)	0.0296 (3)	0.0039 (2)	0.0086 (2)	0.0057 (2)
S2	0.0273 (3)	0.0336 (3)	0.0300 (3)	0.0057 (2)	0.0152 (2)	0.0046 (2)
O1	0.0404 (9)	0.0485 (11)	0.0299 (9)	0.0037 (8)	0.0181 (7)	0.0084 (8)
O2	0.0251 (8)	0.0323 (9)	0.0348 (9)	0.0061 (7)	0.0143 (6)	0.0034 (7)
N1	0.0206 (8)	0.0250 (9)	0.0262 (9)	−0.0011 (7)	0.0099 (7)	−0.0010 (8)
N2	0.0206 (8)	0.0252 (9)	0.0282 (9)	0.0001 (7)	0.0096 (7)	−0.0013 (8)
C1	0.0287 (11)	0.0313 (12)	0.0272 (11)	0.0004 (10)	0.0089 (9)	0.0011 (10)
C2	0.0228 (10)	0.0296 (12)	0.0260 (10)	−0.0002 (9)	0.0087 (8)	0.0003 (10)
C3	0.0221 (10)	0.0253 (11)	0.0245 (10)	−0.0006 (8)	0.0085 (8)	−0.0029 (9)
C4	0.0220 (10)	0.0239 (10)	0.0237 (10)	−0.0003 (8)	0.0083 (8)	−0.0029 (9)
C5	0.0202 (10)	0.0262 (11)	0.0251 (10)	−0.0015 (9)	0.0081 (8)	−0.0037 (9)
C6	0.0200 (10)	0.0228 (10)	0.0260 (10)	−0.0005 (8)	0.0065 (8)	−0.0038 (9)
C7	0.0268 (10)	0.0262 (11)	0.0269 (11)	−0.0030 (9)	0.0096 (8)	−0.0009 (9)
C8	0.0293 (11)	0.0238 (11)	0.0277 (11)	0.0007 (9)	0.0048 (9)	0.0018 (9)
C9	0.0255 (11)	0.0278 (12)	0.0340 (12)	0.0065 (9)	0.0067 (9)	−0.0016 (10)
C10	0.0223 (10)	0.0315 (12)	0.0287 (11)	0.0016 (9)	0.0088 (8)	−0.0022 (10)
C11	0.0226 (10)	0.0243 (10)	0.0247 (10)	−0.0009 (8)	0.0077 (8)	−0.0021 (9)
C12	0.0226 (10)	0.0282 (11)	0.0357 (12)	−0.0010 (9)	0.0173 (9)	0.0010 (10)
C13	0.0362 (12)	0.0401 (14)	0.0366 (13)	−0.0039 (11)	0.0215 (10)	−0.0032 (11)

### Geometric parameters (Å, º)

**Table:**

S1—C2	1.724 (2)	C6—C11	1.411 (3)
S1—C1	1.778 (2)	C7—C8	1.378 (3)
S2—C3	1.755 (2)	C7—H7	0.9500
S2—C1	1.758 (2)	C8—C9	1.395 (3)
O1—C1	1.212 (3)	C8—H8	0.9500
O2—C5	1.232 (3)	C9—C10	1.371 (3)
N1—C5	1.370 (3)	C9—H9	0.9500
N1—C6	1.392 (3)	C10—C11	1.403 (3)
N1—C12	1.475 (3)	C10—H10	0.9500
N2—C4	1.296 (3)	C12—C13	1.519 (3)
N2—C11	1.374 (3)	C12—H12A	0.9900
C2—C3	1.341 (3)	C12—H12B	0.9900
C2—H2	0.95 (2)	C13—H13A	0.9800
C3—C4	1.465 (3)	C13—H13B	0.9800
C4—C5	1.483 (3)	C13—H13C	0.9800
C6—C7	1.399 (3)		
			
C2—S1—C1	96.00 (11)	C6—C7—H7	120.1
C3—S2—C1	95.93 (11)	C7—C8—C9	121.1 (2)
C5—N1—C6	122.51 (18)	C7—C8—H8	119.4
C5—N1—C12	117.57 (17)	C9—C8—H8	119.4
C6—N1—C12	119.91 (18)	C10—C9—C8	119.9 (2)
C4—N2—C11	119.18 (18)	C10—C9—H9	120.1
O1—C1—S2	123.50 (19)	C8—C9—H9	120.1
O1—C1—S1	123.48 (19)	C9—C10—C11	120.2 (2)
S2—C1—S1	113.02 (13)	C9—C10—H10	119.9
C3—C2—S1	118.16 (17)	C11—C10—H10	119.9
C3—C2—H2	124.3 (15)	N2—C11—C10	118.85 (19)
S1—C2—H2	117.4 (15)	N2—C11—C6	121.32 (19)
C2—C3—C4	129.5 (2)	C10—C11—C6	119.8 (2)
C2—C3—S2	116.86 (17)	N1—C12—C13	112.79 (18)
C4—C3—S2	113.64 (15)	N1—C12—H12A	109.0
N2—C4—C3	115.80 (19)	C13—C12—H12A	109.0
N2—C4—C5	124.1 (2)	N1—C12—H12B	109.0
C3—C4—C5	120.11 (19)	C13—C12—H12B	109.0
O2—C5—N1	121.94 (19)	H12A—C12—H12B	107.8
O2—C5—C4	123.6 (2)	C12—C13—H13A	109.5
N1—C5—C4	114.42 (18)	C12—C13—H13B	109.5
N1—C6—C7	122.65 (19)	H13A—C13—H13B	109.5
N1—C6—C11	118.2 (2)	C12—C13—H13C	109.5
C7—C6—C11	119.16 (19)	H13A—C13—H13C	109.5
C8—C7—C6	119.8 (2)	H13B—C13—H13C	109.5
C8—C7—H7	120.1		
			
C3—S2—C1—O1	−178.3 (2)	N2—C4—C5—N1	−4.3 (3)
C3—S2—C1—S1	1.57 (15)	C3—C4—C5—N1	174.71 (18)
C2—S1—C1—O1	179.1 (2)	C5—N1—C6—C7	175.2 (2)
C2—S1—C1—S2	−0.82 (15)	C12—N1—C6—C7	−3.6 (3)
C1—S1—C2—C3	−0.6 (2)	C5—N1—C6—C11	−4.9 (3)
S1—C2—C3—C4	−176.09 (18)	C12—N1—C6—C11	176.29 (19)
S1—C2—C3—S2	1.9 (3)	N1—C6—C7—C8	178.9 (2)
C1—S2—C3—C2	−2.1 (2)	C11—C6—C7—C8	−0.9 (3)
C1—S2—C3—C4	176.21 (16)	C6—C7—C8—C9	0.3 (3)
C11—N2—C4—C3	−178.92 (19)	C7—C8—C9—C10	0.2 (4)
C11—N2—C4—C5	0.1 (3)	C8—C9—C10—C11	0.0 (3)
C2—C3—C4—N2	178.4 (2)	C4—N2—C11—C10	−179.0 (2)
S2—C3—C4—N2	0.4 (3)	C4—N2—C11—C6	1.9 (3)
C2—C3—C4—C5	−0.7 (4)	C9—C10—C11—N2	−179.8 (2)
S2—C3—C4—C5	−178.67 (16)	C9—C10—C11—C6	−0.7 (3)
C6—N1—C5—O2	−174.7 (2)	N1—C6—C11—N2	0.3 (3)
C12—N1—C5—O2	4.1 (3)	C7—C6—C11—N2	−179.8 (2)
C6—N1—C5—C4	6.6 (3)	N1—C6—C11—C10	−178.7 (2)
C12—N1—C5—C4	−174.59 (18)	C7—C6—C11—C10	1.2 (3)
N2—C4—C5—O2	177.0 (2)	C5—N1—C12—C13	−96.3 (2)
C3—C4—C5—O2	−4.0 (3)	C6—N1—C12—C13	82.5 (3)

### Hydrogen-bond geometry (Å, º)

**Table:**

*D*—H···*A*	*D*—H	H···*A*	*D*···*A*	*D*—H···*A*
C2—H2···O2	0.95 (2)	2.30 (3)	2.859 (3)	117.2 (19)
C2—H2···O2^i^	0.95 (2)	2.44 (2)	3.133 (3)	129 (2)
C7—H7···O1^ii^	0.95	2.51	3.272 (3)	138
C9—H9···S2^iii^	0.95	2.99	3.652 (3)	128
C12—H12*A*···O2^iv^	0.99	2.69	3.367 (3)	126

Symmetry codes: (i) −*x*, −*y*+2, −*z*+1; (ii) *x*, −*y*+1, *z*+1/2; (iii) −*x*+1/2, −*y*+1/2, −*z*+1; (iv) *x*, *y*−1, *z*.
